# Human Activities and Postures Recognition: From Inertial Measurements to Quaternion-Based Approaches

**DOI:** 10.3390/s19194058

**Published:** 2019-09-20

**Authors:** Makia Zmitri, Hassen Fourati, Nicolas Vuillerme

**Affiliations:** 1 GIPSA-Lab, Department of Automatic Control, University Grenoble Alpes, 38000 Grenoble, France; makia.zmitri@gipsa-lab.fr; 2 AGEIS, Univ. Grenoble Alpes, 38000 Grenoble, France; nicolas.vuillerme@univ-grenoble-alpes.fr; 3 Institut Universitaire de France, 75231 Paris, France

**Keywords:** activity recognition, wearable sensors, raw data, attitude estimation, subspace KNN

## Abstract

This paper presents two approaches to assess the effect of the number of inertial sensors and their location placements on recognition of human postures and activities. Inertial and Magnetic Measurement Units (IMMUs)—which consist of a triad of three-axis accelerometer, three-axis gyroscope, and three-axis magnetometer sensors—are used in this work. Five IMMUs are initially used and attached to different body segments. Placements of up to three IMMUs are then considered: back, left foot, and left thigh. The subspace k-nearest neighbors (KNN) classifier is used to achieve the supervised learning process and the recognition task. In a first approach, we feed raw data from three-axis accelerometer and three-axis gyroscope into the classifier without any filtering or pre-processing, unlike what is usually reported in the state-of-the-art where statistical features were computed instead. Results show the efficiency of this method for the recognition of the studied activities and postures. With the proposed algorithm, more than 80% of the activities and postures are correctly classified using one IMMU, placed on the lower back, left thigh, or left foot location, and more than 90% when combining all three placements. In a second approach, we extract attitude, in term of quaternion, from IMMUs in order to more precisely achieve the recognition process. The obtained accuracy results are compared to those obtained when only raw data is exploited. Results show that the use of attitude significantly improves the performance of the classifier, especially for certain specific activities. In that case, it was further shown that using a smaller number of features, with quaternion, in the recognition process leads to a lower computation time and better accuracy.

## 1. Introduction

Human activity recognition (HAR) has been a topic of broad and current interest of so many researchers in different applications primarily dealing with human-centric problems such as health [1], fitness [2], elderly care [3], surveillance-based security [4], or context-aware computing [5]. HAR deals basically with the integration of sensing and reasoning in order to identify activities such as walking or sitting, which provides useful feedback regarding individual’s behavior. For instance, in medical applications, patients with diabetes, neural, or heart problems are required to follow a well-defined exercise routine as part of their treatment and recovering process [6].

Over the past decades, a significant development in microelectronics has been witnessed enabling sensors with handy characteristics (small size, low cost, and high computational power) to be exploited in research areas like HAR, where extracting knowledge from the data acquired by these sensors can be very fruitful [7]. The common two modalities in this field are the ones based on external and internal sensing. On one hand, devices can be placed at specific predetermined positions such as cameras, where the detection of activities is fully dependent on the interaction of the user with these devices. Videos and images are the main source of information in this case and computer vision techniques are employed for decision making [8]. There are numerous limitations associated with this method. For example, if the user is not in the range of cameras, movements cannot be detected. Moreover, installation and maintenance of vision equipment entails high costs. In addition, video processing algorithms are computationally expensive since they require a lot of time and memory allocation, which makes real-time HAR system less practical. On the other hand, sensors can be directly attached to the user where there is a guarantee for all-time data collection as the case for inertial and magnetic sensors. In the case of inertial and magnetic sensors-based HAR, the designed system must be able to recognize human activities and postures using information acquired from accelerometers [9,10,11], gyroscopes, magnetometers, or their combination, i.e., HAR with inertial and magnetic measurement units (IMMUs) [12,13,14].

Given the tremendous growth in popularity of smartphones, tablets, and wearable devices, which are always equipped with inertial and magnetic sensors, sensor-based HAR can be very intriguing especially with considering the potential to provide innovative ways of understanding human behavior, as people hold their mobile devices or wear their smartwatches for most of the day.

Although such sensors could reliably measure body segments orientation and movement, recognizing specific patterns remains an open challenge. The most common approaches employed to solve this issue are based on extracting time and/or frequency domain features from the sensors data and then feeding such features to some machine learning algorithms. Statistical measures are mainly considered as time-domain features [15] (mean, standard deviation, root mean square, norm, histograms, etc.), while frequency-domain features are based on the Fourier transform. This feature extraction step can sometimes be inefficient as loading databases with too many attributes can slow down the learning process and lead to high computation cost (159 features in [16] for example).

Moreover, focusing on statistical features only can over shade the physical significance of data and thus provide us with lower detection accuracy rates. As a matter of fact, numerous studies have been done on this matter, in [17], it has been shown that 112 features, extracted from the accelerometer and the gyroscope, are considered important; however, for specific applications they can be lowered to 19 features for the accelerometer and 23 for the gyroscope. For arm and hand side classifications, accelerometer features can be reduced to 4 or even 1. A similar study has been conducted also in [18]. In [19], a comparison has been made between the use separately of angular velocity and linear acceleration features, or their combination. Seven features (time and frequency domain features) were extracted from such measurements. The obtained results prove the pertinence of each case (angular velocity or linear acceleration features) depending on the targeted event.

In this paper, the proposed approach enables the use of rather a raw set of features, instead of time and frequency domain features. It takes into account raw data from not only the gyroscope and the accelerometer, but also the magnetometer, and by combining them, we estimated the attitude, considered later as a new four features (for quaternion) and three features (for Euler angles). In such a way, we guarantee having a small number of features that are not only rich in physical information but are also applicable to any studied activity. We assess also the effect of the number of inertial sensors and their location placements on recognition of human postures and activities. In that case, it was further shown that using a lower number of features, with quaternion (or Euler angles), in the recognition process leads to a lower computation time and better accuracy in certain activities (up and down stairs). As a machine learning approach, we used the k-nearest neighbors (KNN) algorithm, since it was proven to achieve high classification accuracy in literature [11,13,20]. More specifically, and as discussed in [21], an enhanced version of this algorithm can be employed, called subspace KNN. The random subspace technique has been largely discussed in literature [22,23] and it has been shown effective when it was added to classic classifiers such as the KNN.

The subspace KNN is fed with two different features in this paper. First, we choose to work with only raw data attributes and assess if using data coming out from IMMU’s sensors, without any preprocessing, can help the subspace KNN to achieve efficient classification results. Second, we estimate the attitude (quaternion and Euler angles) of the human member from raw data of IMMU’s sensors. Attitude estimation is an area of research well treated in navigation [24,25] and less in HAR. The quaternion is used now as features for the subspace KNN and significantly improved performance of the classifier. To the best of our knowledge, such features have not been used for this recognition issue and constitute one of the main contributions of this paper. We also discussed the different comparisons that we conducted, varying the number, type of sensors and possible body placements and came out with multiple conclusions.

This paper is organized as follows. Section 2 presents the methodology we followed about the used sensors (and measurements), attitude estimation principle, sensor placement and studied activities/postures, data acquisition, and methods for classification. Section 3 exposes a deep discussion about the results of classification for recognition with raw data and quaternion features as well as a discussion about the computation time and accuracy of the proposed methods. We end the paper with some conclusions and future work in Section 4.

## 2. Methodology for HAR

### 2.1. Sensors and Raw Inertial and Magnetic Measurements

**Figure 1:**
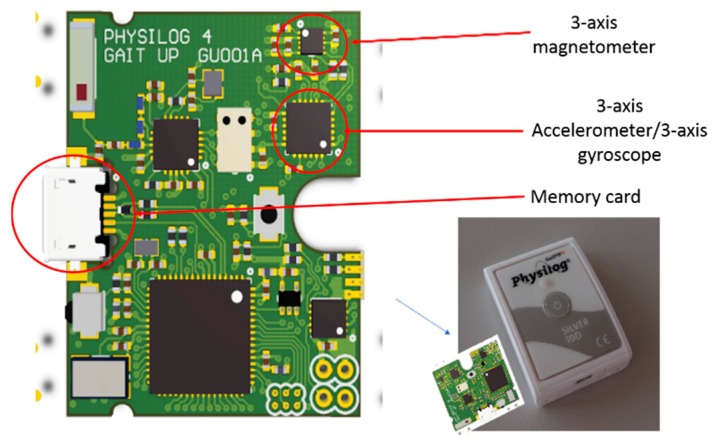
Physilog module from Gait Up.

To achieve our goal related to HAR, we dispose, in the framework of experimental tests, of a set of five wearable modules “Physilog“ from the Gait Up brand [26] (see Figure 1). Each module is a complete miniature IMMU equipped with a triad of three-axis accelerometer, three-axis gyroscope, and three-axis magnetometer, with micro-electro-mechanical systems (MEMS) technology. The raw data recorded from these sensors can be stored on a memory card that equips each module, then used in classification algorithms for further analysis and recognition. The five modules can be synchronized which help us to analyze data from different human limbs and cross the results between them. For the ‘Physilog’ module, the raw data from inertial and magnetic sensors is measured in the sensor’s coordinate system (or body coordinate system) ${ℜ}_{b}\left(\begin{array}{ccc} x_{b} & y_{b} & z_{b} \end{array}\right)$.

A three-axis accelerometer measures the specific force vector $y_{a}\in {ℜ}^{3\times 1}$ (sum of linear acceleration and Earth’s gravity $g=9.81\;m/s^{2}$) and outputs its projection in ${ℜ}_{b}$. A three-axis gyroscope measures the angular velocity vector $y_{g}\in {ℜ}^{3\times 1}$ of ${ℜ}_{b}$. The gyroscope principle uses the Coriolis effect to measure the angular rate. A three-axis magnetometer measures the direction and intensity of the magnetic field, in particular, the Earth’s magnetic field vector $y_{m}\in {ℜ}^{3\times 1}$ in ${ℜ}_{b}$. Usually the outputs of these sensors are corrupted with noise vector $\delta \in {ℜ}^{3\times 1}$ assumed to be a white Gaussian whose components are not correlated.

### 2.2. Attitude Estimation Principle

**Figure 2:**
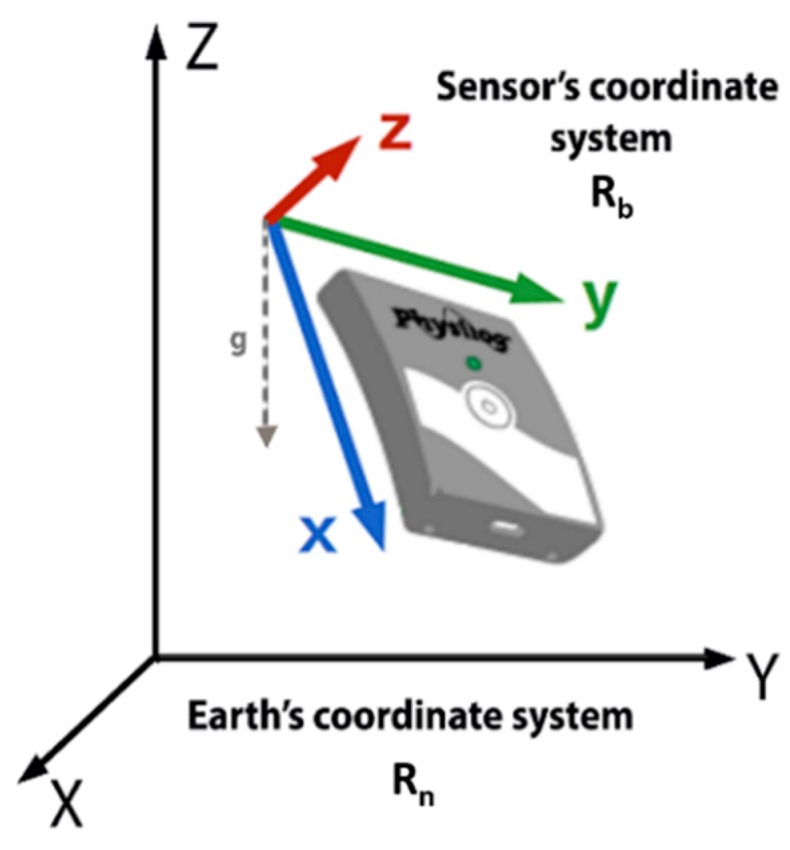
Earth and sensor’s coordinate systems.

In the ‘Physilog’ module, the raw data from sensors is expressed between two coordinate systems (see Figure 2): the sensor’s coordinate systems ${ℜ}_{b}$ and the inertial coordinate system ${ℜ}_{n}\left(\begin{array}{ccc} x_{n} & y_{n} & z_{n} \end{array}\right)$ (considered as the Earth’s coordinate system). The system ${ℜ}_{n}$ is defined according to the NED convention (north, east, down).

Then, we can define the rotation between these two coordinate systems as the attitude of the body segment. To adequately determine the attitude later in the experiments, we make sure that the principal axes of IMMU (composed of the triad of sensors) coincide with those of the body inertia (human limb). The attitude of the body supporting the ‘Physilog’ module can be represented by quaternion or Euler angles. The quaternion, denoted by $Q={\left[\begin{array}{cccc} q_{0} & q_{1} & q_{2} & q_{3} \end{array}\right]}^{T}\in {ℜ}^{4\times 1}$, is a hyper-complex number of rank 4 [27]. Euler angles are defined as a set of three angles (roll: rotation around x-axis, pitch: rotation around y-axis, yaw: rotation around z-axis) [28].

Attitude estimation problem has received a great attention in several areas of application. Not being directly measurable, this information can be reconstructed using estimation algorithms merging measurements from several sensors, depending on the final application. This problem was formulated originally by Wahba [29] and consisted in determining the optimal attitude by using at least two pairs of unit vectors measured in two different coordinate systems, sensor, and Earth ones in our case (${ℜ}_{b}$ and ${ℜ}_{n}$, respectively). A multitude of solutions was proposed to solve this problem, some of first methods are based on deterministic approaches TRIAD [30], QUaternion ESTimator (QUEST) [31], and Singular Value Decomposition method (SVD) [32]. More recently, some dynamic estimation methods, more efficient, such as Kalman filters (KF) [33,34,35] and observers [36,37] are proposed. One of the famous surveys of these methods can be found in [38].

**Figure 3:**
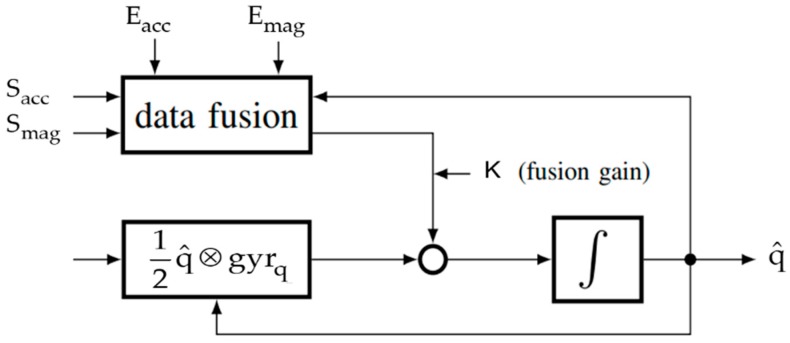
Attitude estimation architecture [39] © 2017 IEEE.

The use of inertial and magnetic sensors has grown these last years on smartphones, tablets, etc. A large number of dynamic estimation methods was implemented on these connected objects. In fact, since the use of only three-axis gyroscope data is not enough for attitude estimation, three-axis accelerometer, and three-axis magnetometer data are added to get an absolute quaternion and compensate the gyro drift from bias. The essence in solving an attitude estimation problem, with dynamic estimation methods, resides in combining such inertial and magnetic sensor measurements in a relevant manner. Figure 3 illustrates the general schema of estimation, where K represents the fusion gain between data that is merged from the accelerometer-magnetometer fusion and the gyroscope integration. This gain is calculated automatically via a specific equation inside KFs, is adjusted depending on sensors reliability for complementary filters, or is calculated from a certain candidate Lyapunov function for observers.

Following this architecture, a typical IMMU can provide two vector observations expressed in two coordinate systems:

1. Acceleration in ${ℜ}_{b}$ provided by a three-axis accelerometer, noted $S_{\mathrm{acc}}$, and its projection in ${ℜ}_{n}$, noted $E_{\mathrm{acc}}={\left[\begin{array}{ccc} 0 & 0 & g \end{array}\right]}^{T}$ (g is the gravity).
2. Earth’s magnetic field in ${ℜ}_{b}$ provided by a magnetometer, noted $S_{\mathrm{mag}}$, and its projection in ${ℜ}_{n}$, noted $E_{\mathrm{mag}}={\left[\begin{array}{ccc} m_{x} & m_{y} & m_{z} \end{array}\right]}^{T}$. $m_{x}$, $m_{y}$, and $m_{z}$ can be obtained using the World Magnetic Model (WMM) [40].

The data fusion block will produce a quaternion that updates the one estimated from three-axis gyroscope data, via the kinematic equation $\frac{1}{2}\hat{q}⊗{\mathrm{gyr}}_{q}$, where ${\mathrm{gyr}}_{q}$ is the quaternion form of angular velocity data.

### 2.3. Sensors Placement and Studied Activities and Postures

Eight healthy subjects, four males and four females, aged between 19 and 46 years, participated voluntarily in this study. Each participant signed written informed consent before the measurement and the ethic approval is obtained. The characteristics of the volunteers are displayed in Table 1.

We provided each subject with the five synchronized ‘Physilog’ modules:

- two modules placed on both feet,
- two modules on the thighs, and
- one module on the lower back.

Since experiments with the five ‘Physilog’ modules show that the combination of both right and left (feet and thighs) does not contribute significantly to the classification accuracy (0.03% of improvement), we chose to present the results from three module locations: left foot (LF), left thigh (LT), and lower back (LB), as they present the most significant placements for the studied postures and activities. The choice of right or left limb is done arbitrarily since no differences are observed between them. The sensors were very securely attached to the participant’s body limbs using a special straps provided by Gait Up. These straps avoid misalignment between the wearable sensors axes and those of the associated body member while recording the different protocols. We have also consulted experts in biomechanics, and we have done some comparisons of sensors outputs with a motion capture system localized in a special room equipped with Vicon and OptiTrack cameras, to make sure they are fixed on the body, in the most convenient way with the minimum error of alignment.

**Figure 4:**
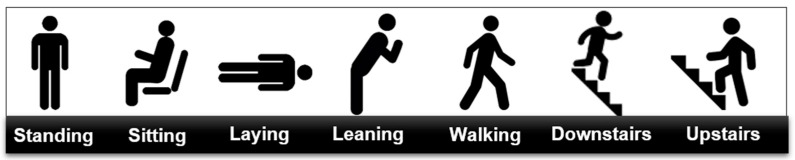
Studied activities and postures.

The subjects were given instructions to perform activities and postures in their own way without specific constraints, however we asked them to follow certain protocols where the order and the duration of the performed activities were specified. No restrictions have been made on the clothes or shoes worn by the participants (sneakers, boots, heels, etc.). Each subject conducted a test scenario composed of three different protocols that are performed separately, giving us in total, seven activities/postures to classify: standing, sitting, laying, leaning, walking, downstairs and upstairs (see Figure 4).

The eight subjects were asked to follow a certain predefined order and duration of the proposed activities. This has enabled us later to accurately label the training data by knowing the start and the end times of each activity or posture. The three conducted protocols are detailed in Table 2. Some of the performed activities or postures are added for synchronization or labeling reasons, such as ‘jumping jacks’, ‘wait’, and ‘turn’. They play a huge role in the detection of the targeted seven postures/activities as they help us distinguish them from each other, especially when two activities are performed successively and are both static or dynamic. For instance, during labeling, the jumping jack activity enables us to differentiate between static activities, such as sitting and standing up, as they have very similar raw signals, while the jumping jack in between these two postures is highly dynamic, and thus it helps us detect the end of sitting and the start of standing up (check first protocol in Table 2). Similarly, the ‘turn + wait’ activity is performed to recognize going up stairs from going down stairs (third protocol). This manipulation is done during the data preparation phase in order to have a clean and correctly labeled training dataset.

### 2.4. Data Acquisition and Preparation

**Figure 5:**
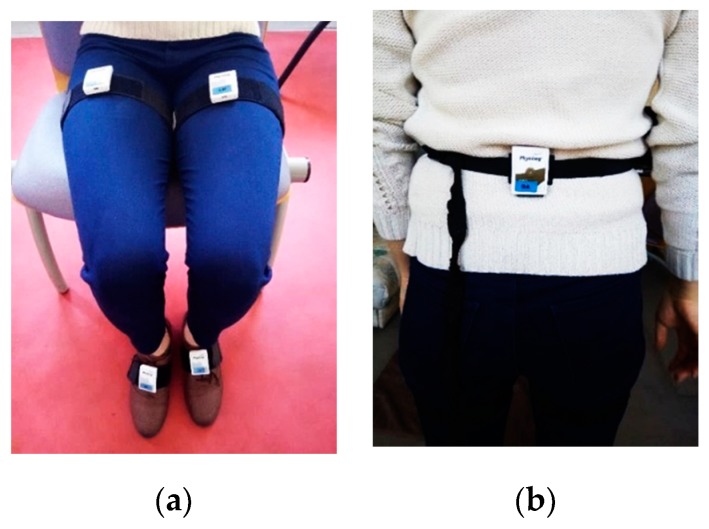
(**a**) Four modules placed on thighs and feet; (**b**) One module placed on the lower back.

The data acquisition was performed in a reproduced apartment environment over a period of about three hours in total. The apartment was composed of chairs, a sofa, a bed, a desk, some home appliances (TV, coffee machine, food mixer, etc.), a long hallway, stairs and elevators, which enabled us to conduct all the studied activities in a very natural manner. No large magnetic perturbations were observed in this environment despite the fully realistic environment. A deeper study of high magnetic disturbances effect should be performed, but is not in the objectives of this paper. As discussed earlier, experiments have been done on five possible locations that are displayed in the Figure 5. Sensor configurations have been made in order to set the range, the units and the sampling frequency (50 Hz). This was through the ‘Physilog Research Toolkit’ (RTK). To synchronize the recordings of the different modules, we identified one sensor as a master and the others as slaves as recommended by the manufacturer [26]. It remains mandatory though to make sure to switch them ON at the same time in order to have a perfect synchronization, which is not easy to achieve each time and calls for a pre-treatment phase of the collected databases as mentioned in the previous paragraph. As we have applied three specific protocols for the seven activities, then we know exact information on when each activity should start and how much time it should last. This has enabled us to subtract samples that largely exceeded the expected number of samples for each activity (and for each IMMU) and to ensure the synchronization between different IMMUs.

Raw data is recorded and stored in the IMMU’s memory card, then extracted using the RTK and organized in a ‘.csv file’ that is later converted into an “Excel file” for easier manipulation. As we chose to be in the supervised machine learning framework, it was necessary to manually label our acquired raw data with the appropriate class (activity/posture). This phase is very critical to the efficiency of our proposed approach, as it affects directly the training class and can cause false recognition if some samples are mislabeled. This may occur mainly because of the uneven number of samples between the different collected databases, caused by delays and slight unsynchronized recordings of the different sensors. A pre-treatment phase is necessary to subtract beginnings and ends of raw data in order to have synchronized databases for all sensors from each module. In addition, false labeling is likely to happen in the areas of transition between two successive activities.

This is why we highlighted these transitions with the ‘jumping jack’ and ‘wait and turn’ activities, as discussed in Section 2.4. Since we are not interested in studying these transitions further, we have eliminated them from the different recordings.

### 2.5. Overview of the Proposed Approach for HAR

**Figure 6:**
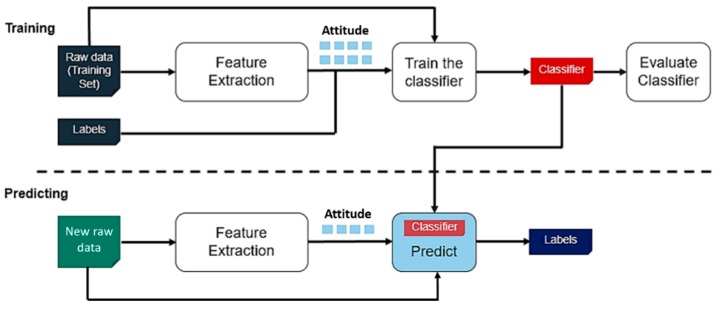
Proposed approaches for HAR with raw data or attitude.

The proposed methodology for HAR process begins with the real world in which each wearable ‘Physilog’ module is affixed to a person’s body segment. First, the training step consists in collecting raw data from sensors (for each module) after sampling the signals, for a training scenario. In a first approach, this raw data is used as features to train the classifier (form the training set). We recall that the data is not preprocessed or filtered beforehand. In a second approach, the raw data is used to extract features (quaternion or Euler angles) through an attitude estimation algorithm (see Section 3.3). Second, the predicting step consists in exploiting new observations to effectively associate them to their corresponding class by using the chosen classifier (already trained). The overall method is illustrated in Figure 6.

The main goal of the classification process is to allocate an object represented by a number of measurements (i.e., feature vectors) into one of a finite set of classes. In order to do so, a number of training samples are available for each class, and they are used to train the classifier. When new data is available, whether it is kept as raw or transformed into attitude features, the classifier tries to predict its corresponding output using a learned function. This falls into the supervised learning category in pattern recognition. K-nearest neighbors (KNN) [41] is considered as one of the simplest and most effective algorithms for achieving our objective. An advanced version of this algorithm, the subspace KNN, is used in this work, a combination between the KNN algorithm, and the random subspace technique. To estimate the effectiveness of the classifier, a validation technique needs be adapted to test the accuracy of the recognition model when a new data arrives.

To evaluate the efficiency of the proposed approach in this paper, we have used the leave-one-out cross validation, which means that we learn about n − 1 observations, and then validate the model on the umpteenth observation, and repeat this operation n times. As we are working with eight subjects, the algorithm has been executed eight times, where each time a different subject is considered in the testing and the other seven subjects are for the training. Accuracy results for the eight executions were very similar. For this reason, we display on the paper the results of one execution that is arbitrarily chosen.

#### 2.5.1. K-Nearest Neighbors Algorithm

**Figure 7:**
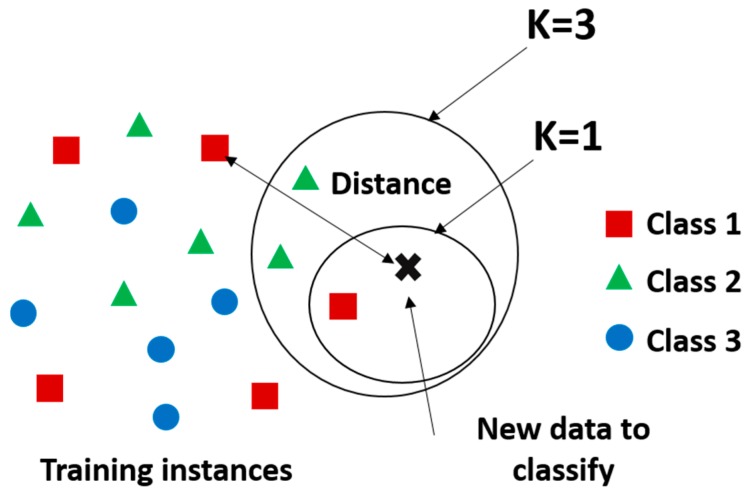
Example of KNN classification. The test sample (cross) should be classified to one of the three classes. If K = 3 (outside circle), it is assigned to the second class because there are two triangles and only one square inside the inner circle.

The KNN algorithm is a supervised learning algorithm that is instance-based and non-parametric. With instance-based we mean that the function is only approximated locally and all computation is achieved until a prediction is required. In other words, there is no explicit training phase and training data points are not used to do any generalization. KNN is also non-parametric, which applies that it does not make any assumptions on the underlying data distribution. Thus, the model structure is determined from the data itself. KNN algorithm is based on feature similarity, more specifically, it identifies how closely new features of a given data point resemble to those of the training set, in order to affect that point to its corresponding class, such as demonstrated in Figure 7. In this context, KNN performs a majority vote between the K most similar instances to a new unseen observation. This similarity is defined according to a calculated distance between two data points. One of the most used distance metrics is the Euclidean distance given by

(1) $$d\left(x,x^{\prime }\right)=\sqrt{{\left(x_{1}-{x^{\prime }}_{1}\right)}^{2}+{\left(x_{2}-{x^{\prime }}_{2}\right)}^{2}+\ldots +{\left(x_{n}-{x^{\prime }}_{n}\right)}^{2}}$$

Therefore, given a positive integer K, a new data $x^{\prime }$ and a similarity metric d, KNN achieves the following two steps:

- first it calculates d between $x^{\prime }$ and each training sample,
- then it estimates the conditional probability for each class by
  (2) $$P\left(y=j|X=x^{\prime }\right)=\frac{1}{K}\sum_{i\in A}I\left(y^{\left(i\right)}=j\right)$$
  where A is the set that contains the K points in the training data that are closest to $x^{\prime }$, I(x) is the indicator function which equals 1 when the argument x is true and 0 otherwise. Finally, our input $x^{\prime }$ gets assigned to the class with the largest probability.

The choice of the K value to be used varies according to the dataset. As a rule, the fewer neighbors (a small number K) we have, the more we are subject to under-fitting. Using more neighbors (a large K number) is then more reliable for the prediction. However, if we use K = N number of neighbors, with N being the number of observations, we risk causing overfitting and consequently a model that generalizes badly on observations that it has not seen yet. It is then mandatory to select the optimal value of K for the given training set, by running the classification algorithm several times with different values of K (from 1 to 30), until the best classification accuracy result of a new upcoming data (testing set) is obtained. In our case, the optimal value of K was equal to 5.

#### 2.5.2. Subspace KNN Algorithm

As its name suggests, the subspace KNN is a method that combines the KNN algorithm described above and the random subspace (RS) technique. The RS algorithm [42] is an ensemble learning method that tries to reduce the correlation between different learners by training them on random samples of features, with replacement (a feature can be selected more than one time), instead of the entire feature set. This helps these individual learners avoid over focusing on features that seem highly descriptive in the training set, but are in fact less predictive for points outside the set.

The subspace KNN can be constructed using the following algorithm:

- Let N be the length of the training set, D be the number of its features and L be the number of individual models in the ensemble.
- For each individual model l, we choose n_l_ (n_l_ < N) to be the number of input points for l. It is common to have only one value of n_l_ for all individual models.
- For each individual model l, we create a training set by selecting d_l_ features from D with replacement and train the model.
- Finally, we combine the outputs of the L individual models using the majority vote. This vote outputs the class that has been chosen the most from all the different subspaces. The advantage of this method is that it enables us to have a different result for each subspace coming from the main training set, to later select the most recurrent one. This has been proven beneficial to avoid issues like overfitting [21,42].

**Figure 8:**
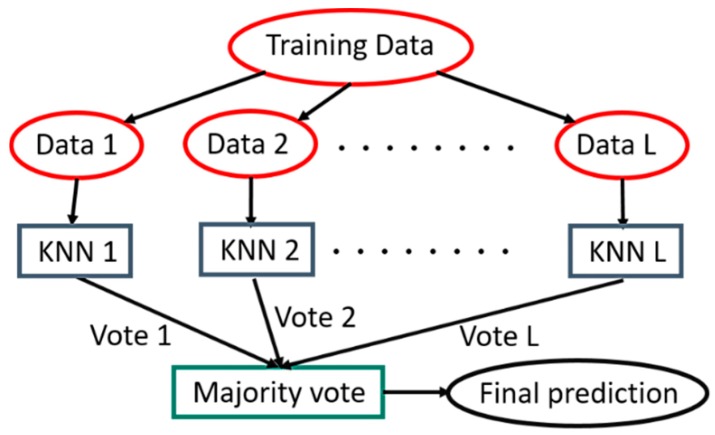
Subspace KNN architecture.

To apply this algorithm, we first create L random samples, with replacement, of a given size n_l_, from the training set, and then we compute a single KNN classifier for each sample, as shown in Figure 8. Next, we will get a vote from each classifier about the correspondence of a new instance to a particular class in order to determine, through the majority vote, the final prediction.

## 3. Classification Results and Discussion for HAR

### 3.1. Confusion Matrix for Evaluation of Classification’s Performance

**Figure 9:**
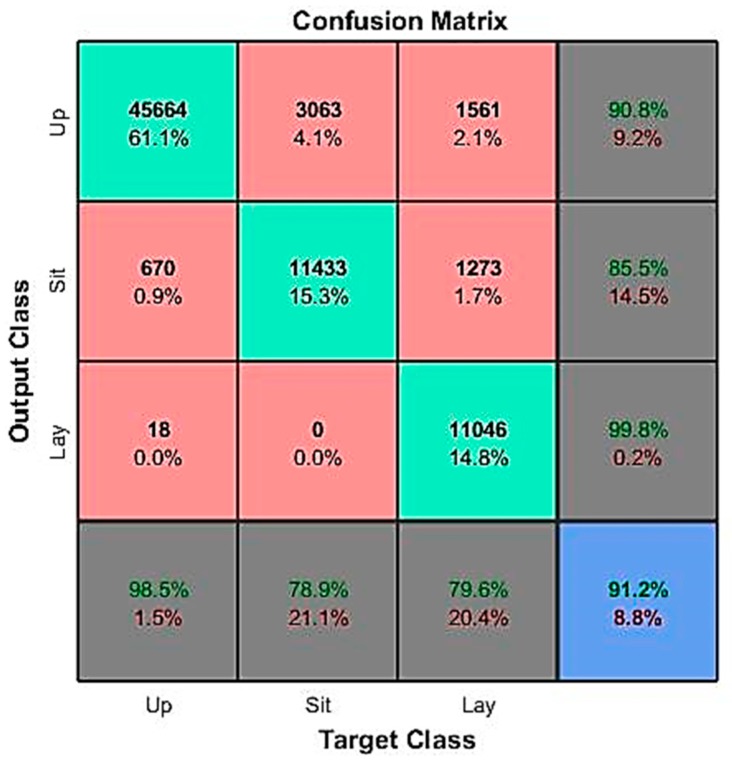
A confusion matrix example.

A confusion matrix is a specific table layout that enables visualization of the performance of a recognition model (or ‘classifier’) on a set of test data for which the true values are known. It has two dimensions (‘actual’ and ‘predicted’), and identical sets of ‘classes’ in both dimensions. In Figure 9 for example, the rows correspond to the predicted class (output class) and the columns correspond to the true class (target class). The diagonal cells represent observations that are correctly classified, while the off-diagonal cells are incorrectly classified observations. Both the number of observations and the percentage of the total number of observations are displayed in each cell. The column on the far right shows the percentages of all examples predicted to belong to each class that are correctly and incorrectly classified. Usually, these metrics are referred to as the ‘accuracy’ (or positive predictive value) and ‘false discovery rate’, respectively. On the other hand, the row at the bottom shows the percentages of all the examples belonging to each class that are correctly and incorrectly classified. We call these metrics the ‘recall’ (or ‘true positive rate’) and ‘false negative rate’, respectively. The cell in the bottom right corner is the overall accuracy of the algorithm, which represents the accuracy that we show in all our results and interpretations.

In Figure 9, the three diagonal cells show the number and percentage of correct classifications by the trained subspace KNN. For example, 45,664 samples are correctly classified as the standing activity. This corresponds to 61.1% of all 74,728 samples. Similarly, 11,046 cases are correctly classified as the laying activity. This corresponds to 14.8% of all samples. 670 of the standing samples are incorrectly classified as sitting and this corresponds to 0.9% of all data. Similarly, 1561 of the laying samples are incorrectly classified as the standing position and this corresponds to 2.1% of all data. Out of 46,352 standing cases, 98.5% are correctly predicted as standing and 1.5% are wrongly predicted as sitting and laying. Out of 50,288 standing predictions, 90.8% are correct and 9.2% are mistaken. Overall, 91.2% of the predictions are correct and 8.8% are false.

### 3.2. Results for Subspace KNN Algorithm with Raw Inertial Data Features

**Figure 10:**
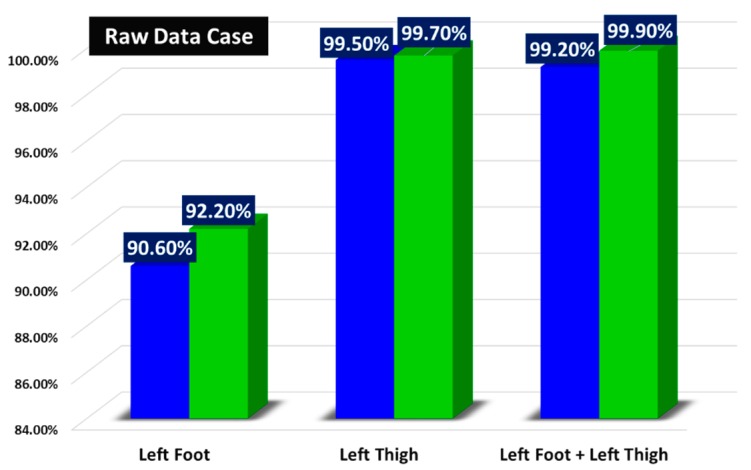
Comparison between the use of three-axis accelerometer raw data and the addition of three-axis gyroscope one in the case of static activities.

It is well known that adding redundant or excessive data (features) will not only slow down the learning process but can also mislead the classification procedure. In fact, the predictions of instance-based methods such as KNN, that uses small neighborhoods in the attribute space, can be greatly skewed by redundant attributes. This is the reason why it is crucial to perform feature selection where we automatically search for the best subset of attributes in the dataset. To support this claim, a comparison is made between the use of only three-axis accelerometer data and the addition of data from three-axis gyroscope. By conducting this experiment, we aim to see if adding redundant features to the training set (three-axis data of gyroscope) will provide us with noticeable improvement in recognition accuracy compared to when we use just the three-axis data coming from the accelerometer. As a first step, the comparison is done on three postures (sitting, standing, and laying), we focused on three different location scenarios (LF, LT, and their combination) for simplicity reasons. What we can conclude from Figure 10 is that accelerometer data, no matter which position it is recorded in, can be considered enough to obtain high recognition accuracy results (more than 90% in all three location scenarios) in the case of static activities. The addition of gyroscope data does not improve significantly these results (1.6% and 0.2% for the left foot and left thigh locations respectively), and thus as a compromise, to have a faster learning process and less memory usage, it can be not taken into account.

As mentioned earlier in this paper, we noticed that almost all the related work employ statistical features for the recognition process such as norm, standard deviation, mean, etc. Usually, these works report high accuracy results. We wanted to verify this statement and the efficiency of this approach by implementing the same experiment described above and using norms instead of raw data.

**Figure 11:**
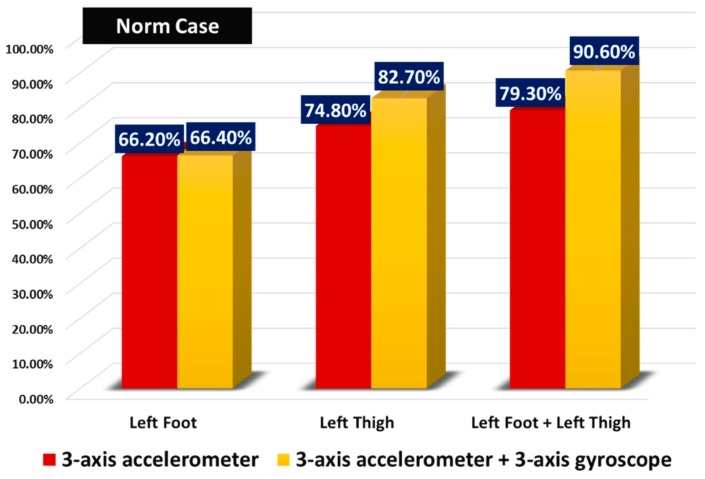
Recognition accuracies of static activities when using the norm of raw data as a feature.

In Figure 11, we notice a remarkable decrease in the recognition accuracy, compared to results given by raw data in Figure 10, which was actually expected as moving to norms over shades information from each axis of sensors and that can be crucial for the recognition of some activities. This proves that raw data remains more interesting to use rather than transforming it into statistical features. It is noticed however that three-axis gyroscope data has now better contribution in the recognition accuracy (7.9% of improvement in accuracy for the left thigh case), as by calculating the norm of the three-axis values of the accelerometer, we will have only one feature with which the KNN classifier can learn, and thus it cannot effectively distinguish the three activities, but by adding one additional norm that comes from the three-axis gyroscope (having in total two norms representing the features), the learner is able to have a better performance. The same results are found for other activities (walking, downstairs and upstairs), where gyroscope data enhances the classification accuracy, thus, for the raw data case, we proceed with six features coming from a three-axis accelerometer and a three-axis gyroscope.

We repeated the same experiment described in Figure 10, rather by working now on the rest of dynamic activities, to have in total seven activities and postures as mentioned before. In Table 3, we have introduced more possible location scenarios and we compared the obtained classification accuracy in each case. We can clearly notice that combining the different studied locations gives the best accuracy. This result was expected as each location is more suited to classify a particular activity. For instance, having a sensor on the foot will enable us to differentiate sitting from walking, but cannot recognize precisely walking from going upstairs as both activities have very close feet dynamic. However, adding a sensor on the thigh or back can definitely help better determine the difference between these two activities. As a matter of fact, one should point out the pertinence of working with the lower back location, as it presents to some extent, the center of gravity of the human body, and thus it is capable of effectively detect most of the performed activities. Results show that the LB position can provide high accuracy (80.1%), which is very convenient, especially from a customer point of view, because placing a sensor on the back is more user friendly, and easy to work with. Applications in the medical field or sports need flexibility and thus compromises must be done. Having a better sensor placement with lower accuracy can then be preferred in certain cases. This is why, in the continuity of this paper, we are going to work only with the LB data.

### 3.3. Results for Subspace KNN Algorithm with Attitude Features

The most classification approaches in literature for HAR are based on inertial data, and sometimes with too many attributes that can slow down the learning process and lead to high computation cost. Since the attitude represents the rotation of body in space, it can be an effective feature for recognition of activities and postures, especially those that are more complex. Two aspects can be targeted with quaternion-based classification: computation time and accuracy.

In this work, we are interested in quaternion and Euler angles that we obtain from raw data provided by the triad of sensors, using an attitude estimation filter. These attitude-based features will represent the only attributes for the recognition process.

#### 3.3.1. Madgwick’s Filter for Attitude Estimation and Features Extraction

In 2010, Madgwick [43] proposed a new algorithm that uses inertial and magnetic measurements from an IMMU and attempts to leverage on these measurements to provide precise attitude estimation for pedestrians. The proposed filter uses a quaternion representation of orientation and is not subject to the problem of singularities associated with Euler angles. Madgwick addressed issues of computational load and parameter tuning associated with Kalman-based approach. The main idea is to use three-axis accelerometer and three-axis magnetometer data in an analytically derived and optimized gradient descent (GD) based algorithm, in order to compute the direction of the gyroscope measurement error as a quaternion derivative. This algorithm also incorporates magnetic distortion and gyroscope bias drift compensation. The main advantages of this filter include the fact that it is computationally inexpensive, as it requires 277 scalar arithmetic operations each filter update, it is efficient at low sampling rates and it has only two adjustable parameters defined by observable system characteristics. For all these reasons and comparing to other approaches in the literature, we implemented this algorithm to achieve the attitude estimation task, for features extraction (see Figure 6). The main architecture of this approach follows the general idea presented in Figure 3. The readers can refer to [43] for more details on this algorithm and the way it is implemented.

#### 3.3.2. Results of Classification with Attitude-Based Features

**Figure 12:**
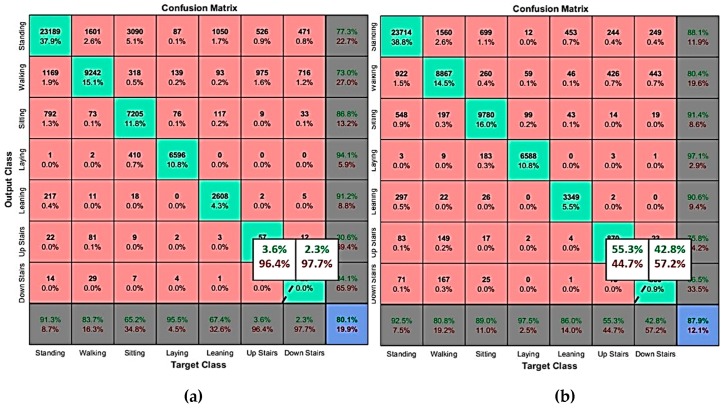
(**a**) Classification with raw data; (**b**) Classification with quaternion.

We have compared activities and postures recognition accuracy obtained when using attitude features to the case with raw data (three-axis accelerometer + three-axis gyroscope data). As cited earlier, we focused on the situation where the IMMU is placed on the lower back. Figure 12 displays the confusion matrices for evaluation of classification in two cases: when employing raw data (Figure 12a) and quaternion (Figure 12b) as features of the process. These results are related to one subject arbitrarily chosen since accuracy results for the eight subjects corresponding to the leave-one-out cross validation techniques were very similar. As presented in Table 3, the overall recognition accuracy using raw data is 80.1%. We notice that the use of quaternion instead, significantly enhances this accuracy by 7.8% (4792 samples in more, well classified compared to the case of classification with raw data), especially for certain relevant activities. For example, the accuracy of the ‘going upstairs’ activity improves from 3.6% with the raw data approach to 55.3% using quaternion one. A similar result is found for the ‘going downstairs’ activity, with an increase of accuracy from 2.3% to 42.8%.

We have conducted the same experiment with Euler angles instead of quaternion. However, results presented in Table 4 do not show a significant improvement in the recognition accuracy compared to when we employ raw data (from 80.1% to 80.3%). Using Euler angles may be interesting as we need only three features (roll, pitch, yaw) to predict the activities, and thus this approach is computationally cheap as it requires the least time and memory. Yet, due to Gimbal lock phenomenon discussed in Section 2.2*,* attitude singularities can make Euler angles unsuited to represent correctly the different activities in some cases and consequently they will produce less accuracy results. Then, we recommend using quaternion for activities recognition.

At the end we want to stress the following two points: (1) the variety in shoes worn by the subjects has not influenced the classification process in our case; (2) Even though our classification approach was tested in a real domestic environment (inside and outside the apartment), where there were elevators, home appliances, iron cupboards, iron desks, iron beds, and so on, our results were not affected by such perturbations. Indeed, the proposed approach for attitude estimation can mitigate such magnetic perturbations as it incorporates a magnetic distortion compensation.

### 3.4. Extended Discussion on Computation Time, Number of Features, and Accuracy

**Figure 13:**
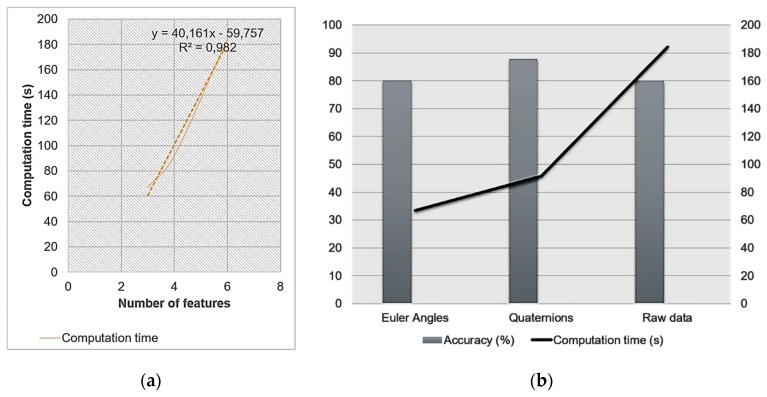
(**a**) Computation time vs. number of features; (**b**) Accuracy vs. computation time.

The main purpose of this work was to achieve human activities and postures recognition using inertial and magnetic measurement. More precisely, we compared recognition’s accuracy when quaternion is used as new features versus when raw inertial data (acceleration and/or angular velocity) is used. Our results show that using quaternion provides better results for some activities recognition (downstairs and upstairs). Now, we can question if using a smaller number of features (four for quaternion) will lead to less computation time? For this matter, a time-based comparison is achieved between the two different recognition approaches, i.e., with raw data or quaternion. MATLAB classification learner toolbox can provide us with information on the time needed for the subspace KNN classifier to learn the training dataset that it has been provided. This enabled us to estimate the learning time of each set of features (raw data or quaternion). We have also used the ‘Tic Toc’ command provided by MATLAB in order to calculate the processing time of each overall algorithm (Data acquisition -> feature extraction -> learning -> prediction -> plotting results). After processing the two proposed approaches, we displayed the results that cross recognition’s accuracy with computation time. For instance, in Figure 13a, we illustrated the evolution of the computation time of classifier when varying the number of used features. As expected, the latter goes higher when employing more attributes, as the training and testing databases will be heavier and their processing is going to be slower. Figure 13b shows a combination between accuracy of the two proposed recognition methods represented by the histograms, and the computation time represented by the curve. What we can conclude here is that using attitude and more specifically quaternion presents the best compromise as it has the highest recognition accuracy and a lower computation time than with raw data.

## 4. Conclusions

In this work, it has been shown that a set of static postures and dynamic activities can be correctly classified by placing only one IMMU on the lower back. Statistical features (such as norm) have given less accuracy results compared to when raw data or attitude features are employed. However, the use of attitude features (three features for Euler angles and four features for quaternion) during the classification process outperforms employing raw data (six features), as it provides the highest accuracy results with the lowest computation cost. The first issue that needs to be addressed in future work is to study the significance of sensors axes in detecting specific activities and see if the same results can be obtained with one-axis (or two-axis) sensors. Secondly, more complex scenarios should be considered in order to quantify the robustness of the proposed approaches.

## Figures and Tables

**Table 1:** Subject characteristics.

Subject	1	2	3	4	5	6	7	8
**Age (years)**	19	33	22	38	46	27	25	23
**Weight (kg)**	53	85	79	86	85	65	90	69
**Height (m)**	1.65	1.77	1.78	1.79	1.82	1.67	1.85	1.58

**Table 2:** Three performed protocols.

Protocol 1	Protocol 2	Protocol 3
Jumping jacks (2 times)	Wait (30 s)	Wait (30 s)
Sitting (1 min)	Walk (1 min)	Go up the stairs (11 steps)
Jumping jacks (2 times)	Wait (30 s)	Turn and wait (30 s)
Standing up (1 min)	Run (1 min)	Go down the stairs (11 steps)
Jumping jacks (2 times)	Wait (30 s)	Turn
Wait (30 s)	Jump (10 times)	Repeat this loop (5 times)
Repeat this loop (5 times)	Repeat this loop (5 times)	
Jumping jacks (2 times)		
Laying on the ground (1 min)		
Jumping jacks (2 times)		
Standing up (1 min)		
Jumping jacks (2 times)		
Wait (30 s)		
Repeat this loop (5 times)		

**Table 3:** Recognition accuracy when using three-axis accelerometer and three-axis gyroscope raw data for different locations.

Locations	LB	LF	LT	LF + LT	LF + LT + LB
Accuracy (%)	80.1	89.9	88.6	92.2	92.9

**Table 4:** Recognition accuracy with raw data, Euler angles, and quaternion in the case of lower back position of IMMU.

**Overall Accuracy (%)**	Raw Data	80.1
	Euler Angles	80.3
	Quaternion	87.9
